# Molecular Mechanisms of Ethanol-Induced Pathogenesis Revealed by RNA-Sequencing

**DOI:** 10.1371/journal.ppat.1000834

**Published:** 2010-04-01

**Authors:** Laura Camarena, Vincent Bruno, Ghia Euskirchen, Sebastian Poggio, Michael Snyder

**Affiliations:** 1 Department of Molecular, Cellular and Developmental Biology, Yale University, New Haven, Connecticut, United States of America; 2 Universidad Nacional Autónoma de México, Inst. Inv. Biomédicas, México, D.F., México; 3 Department of Genetics, Stanford Univeristy School of Medicine, Stanford, California, United States of America; Yale University School of Medicine, United States of America

## Abstract

*Acinetobacter baumannii* is a common pathogen whose recent resistance to drugs has emerged as a major health problem. Ethanol has been found to increase the virulence of *A. baumannii* in *Dictyostelium discoideum* and *Caenorhabditis elegans* models of infection. To better understand the causes of this effect, we examined the transcriptional profile of *A. baumannii* grown in the presence or absence of ethanol using RNA-Seq. Using the Illumina/Solexa platform, a total of 43,453,960 reads (35 nt) were obtained, of which 3,596,474 mapped uniquely to the genome. Our analysis revealed that ethanol induces the expression of 49 genes that belong to different functional categories. A strong induction was observed for genes encoding metabolic enzymes, indicating that ethanol is efficiently assimilated. In addition, we detected the induction of genes encoding stress proteins, including *upsA*, *hsp90*, *groEL* and *lon* as well as permeases, efflux pumps and a secreted phospholipase C. In stationary phase, ethanol strongly induced several genes involved with iron assimilation and a high-affinity phosphate transport system, indicating that *A. baumannii* makes a better use of the iron and phosphate resources in the medium when ethanol is used as a carbon source. To evaluate the role of phospholipase C (Plc1) in virulence, we generated and analyzed a deletion mutant for *plc1*. This strain exhibits a modest, but reproducible, reduction in the cytotoxic effect caused by *A. baumannii* on epithelial cells, suggesting that phospholipase C is important for virulence. Overall, our results indicate the power of applying RNA-Seq to identify key modulators of bacterial pathogenesis. We suggest that the effect of ethanol on the virulence of *A. baumannii* is multifactorial and includes a general stress response and other specific components such as phospholipase C.

## Author Summary

*Acinetobacter baumannii* has recently emerged as a frequent opportunistic pathogen. In the presence of ethanol *A. baumannii* increases its pathogenicity towards *Dictyostelium discoideum* and *Caenorhabditis elegans*, and community-acquired infections of *A. baumannii* are associated with alcoholism. Ethanol negatively affects both epithelial cells and alters the bacterial physiology. To explore the underlying basis for the increased virulence of *A. baumannii* in the presence of ethanol we examined the transcriptional profile of this bacterium using the novel methodology known as RNA-Seq. We show that ethanol induces the expression of a phospholipase C, which contributes to *A. baumannii* cytotoxicity. We also show that many proteins related to stress were induced and that ethanol is efficiently assimilated as a carbon source leading to induction in stationary phase of two different Fe uptake systems and a phosphate transport system. Interestingly, a previous study showed that a mutant in the high-affinity phosphate uptake system was avirulent. Our work contributes to the understanding of *A. baumannii* pathogenesis and provides a powerful approach that can be extended to other pathogenic bacteria.

## Introduction

*Acinetobacters* are Gram-negative bacteria that belong to the Moraxellaceae family [1]. The members of the *Acinetobacter* group are metabolically versatile since they can metabolize an important number of compounds such as aliphatic alcohols, some amino acids, decarboxylic and fatty acids, unbranched hydrocarbons, aromatic compounds, mandelate, and *n*-hexadecane. [2]. Moreover, accumulation of wax esters has been described for various *Acinetobacter* species [3]. These features have attracted attention toward several species of the genus given their potential use in the chemical industry.

Recently, *A. baumannii* has emerged as an opportunistic pathogen. Nosocomial and community acquired infections are associated with a wide spectrum of clinical manifestations, including pneumonia (the most frequent pathology associated with this microorganism), urinary tract infections, bacteremia and meningitis [4]-[7]. Furthermore, there has been a recent emergence of multidrug-resistant (MRD) isolates of *A. baumannii* strains resistant to a wide range of antimicrobial drugs such as aminopenicillins, ureidopenicillins, cephalosporins, chloramphenicol, and tetracycline [8],[9]. Indeed, 89% of *Acinetobacter* strains isolated from patients injured in Iraq and Afghanistan were resistant to at least two major classes of antibiotics [10],[11].

So far, lipopolysaccharide (LPS) [12],[13], an outer membrane protein named OmpA [14],[15], the pili [16], and two siderophore mediated iron-acquisition systems [17]–[19] have been proposed as determinants of *A. baumannii* pathogenicity. It is conceivable that additional elements could be involved in the pathogenesis of this bacterium. The complete genome sequences of several isolates of this species revealed the presence of homologues of virulence genes from other pathogens [20]–[23]. Examples include homologues of *luxI* and *luxR* that allow cell-cell communication, genes that encode two-component systems, genes that code for several hydrolytic enzymes, efflux pumps, and genes involved with resistance to antibiotics. However, in most cases, evidence regarding the contribution of each of these elements to *Acinetobacter* pathogenicity is lacking.

It was previously observed, that co incubation of yeast with *A. baumannii* promotes bacterial growth; the molecule responsible for this effect was shown to be ethanol. It was demonstrated that low concentrations of ethanol not only stimulated *A. baumanni* growth but also helped the ability of this bacteria to endure salt stress. Furthermore, in the presence of ethanol *A. baumannii* showed increased pathogenicity towards *C. elegans* [24]. It was subsequently reported that the increased pathogenicity of ethanol-fed *A. baumannii* was also observed when *D. discoideum* was used as a model host [25]. Furthermore, genes associated with *A. baumannii* virulence were identified by insertional mutagenesis; one of the genes identified by this strategy is homologous to *pstC*, which encodes a component of the high affinity phosphate transport system [25]. Interestingly, in some pathogenic bacteria the phosphate regulon appears to be part of a network that controls virulence and a particular stress response (recently reviewed by [26]). Another gene identified in the insertional screen was *rpoH*, which in many species, encodes the sigma factor that is responsible of the transcriptional induction of the genes that mediate the heat-stress response (HSR) [27]. The HSR has been shown to be required for full virulence in other pathogenic bacteria [28]–[31]. Thus, the potential contribution of the HSR to the virulence of *A. baumannii* remains as an attractive possibility.

To explore the underlying basis for the increased virulence of *A. baumannii* in the presence of ethanol, we characterized the transcriptional profile of *A. baumannii* grown in rich medium in the presence or absence of ethanol. Seventy genes whose expression is altered by the presence of ethanol in the growth medium were identified. Based on our results we suggest that the increased virulence of *A. baumannii* in the presence of ethanol is due to increased metabolic capacity, coupled with the expression of several key factors mostly related with stress responses that likely contribute to the virulence of this bacterium. In addition, ethanol promotes the expression of *plc1*, which encodes an *A. baumannii*-specific phospholipase C. We found that a *plc* mutant showed a reduction in *A. baumannii*-induced cytotoxicity of epithelial cells, suggesting that phospholipase C acts as a virulence factor in this bacterium.

## Results/Discussion

### Transcriptional profiling of *A. baumannii* by high throughput RNA sequencing

To identify genes important for an inducible virulence response in *Acinetobacter*, we first search for genes that were differentially expressed in the presence of ethanol. Total RNA was isolated from *A. baumannii* cells grown to mid-log phase in the absence or presence of ethanol (see Methods section). For the first set of samples, the mRNA was selectively enriched through a single step of rRNA depletion. These samples were fragmented and used to obtain cDNA libraries that were sequenced as described in Methods section. A total of 6,441,146 and 6,603,654 28 nt reads were obtained for each library (“no ethanol” and “ethanol”, respectively). Of these, 4,364,106, (no ethanol) and 4,824,047 (ethanol) reads mapped to multiple targets in the genome and only 312,266 (no ethanol) and 546,498 (ethanol) mapped uniquely. Analysis of 1% of the reads that mapped to the genome multiple locations revealed that most were derived from 23S, 16S, and 5S rRNA. In order to obtain a more representative sampling of the coding regions, the libraries were sequenced an additional two times. Additional RNA samples were prepared in duplicate experiments and subjected to three-cycles of rRNA depletion followed by size exclusion to remove small RNAs, but no substantial improvement in the number of unique reads was achieved with this procedure.

Combining all of the sequencing from both experiments produced a total of 3,596,474 unique reads that represent a total of 100,701,272 nucleotides (28 nt per read), a 25.3 fold average coverage of the *A. baumannii* genome. The sequences obtained from these experiments were mapped to the *A. baumannii* genome using the current annotation in Genbank (accession: CP000521 version CP000521.1). As expected, between 72 and 84% of the unique sequences correspond to previously annotated coding regions, whereas the remainder correspond to intercistronic regions, RNA molecules such as the tmRNA, ribonuclease P, 7S RNA, and possible regulatory RNAs, such as the TPP riboswitch. Two previously unannotated genes were also identified. These genes are a putative ferredoxine located downstream of A1S_0845, and a gene located upstream of A1S_2262 that has sequence similarity to SirA.

**Figure 1:**
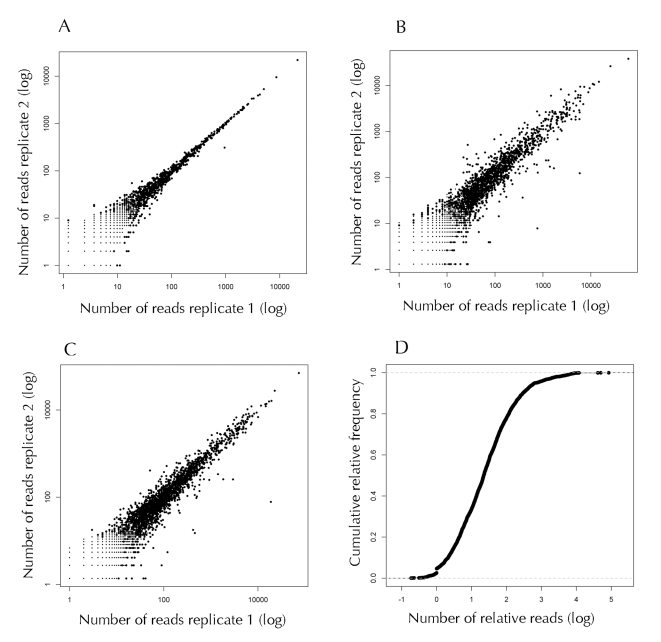
Analysis of the RNA-Seq short sequences (reads) mapped to the genome of *A. baumannii.* (A) Reproducibility between technical replicates. The total reads obtained from independent lanes of one flow cell were mapped to the genome of *A. baumannii.* The number of reads was normalized, and the absolute number of reads mapping to each coding region is compared. (B and C) Reproducibility between biological replicates. The reads obtained from independent libraries were mapped to the genome of *A. baumannii*, the number of reads between libraries was normalized and the absolute number of reads mapping to each coding region is compared. Panel B: Comparison between the libraries obtained from the cultures grown in the absence of ethanol. Panel C: Comparison between the libraries obtained from cultures grown in the presence of 1.1% ethanol. The R-squared values are: panel A, 0.99; panel B, 0.87; panel C, 0.94. (D) Cumulative relative frequency of the number of relative reads (NRR).

Two different approaches were used to demonstrate that the number of reads that map to a particular open reading frame correlate with the expression level of that gene. In the first approach, mRNA from two different tissues was analyzed using microarrays and RNA-Seq [32]. In the second one, microarray and RNA-seq data were compared with protein expression data obtained by shotgun mass spectroscopy [33]. In both cases, good levels of correlation were observed. Therefore, we computed the total number of reads for each gene, and this number was divided into windows of 250 bp to calculate the number of relative reads (NRR) per window. We observed that the highest NRR mapped to the loci corresponding to the tmRNA and RNase P, followed by the genes A1S_2840 (NRR 50,000), and A1S_2218 (NRR 41,000) that encode OmpA (Outer Membrane Protein A), and the pili subunit CsuA/B, respectively. The NRR was also high for genes encoding proteins related to transcription, translation and energy generation. We detected 163 genes with a NRR less than 1 (Table S1), suggesting that some genes are expressed at a low level or that these are not readily detected because of experimental bias. As shown in Figure 1, 50% of *Acinetobacter* genes have NRR values of approximately 150. Both technical and biological duplicates showed high reproducibility (Fig. 1).

To identify the genes that are induced or repressed by ethanol, we normalized the number of reads for each pair of libraries, and the number of reads for each gene was compared. The genes that reproducibly showed a ratio larger than 1.9 or below 0.5 in both biological replicates and a P value of 0.05 or less were considered as regulated by ethanol. From 101 genes that passed the first criterion, only 70 showed a P-value below 0.05. From these, 21 genes were repressed and 49 were induced by ethanol (Table 1).

**Table 1:** Up-regulated and down-regulated genes in *A. baumannii* (Ab) by ethanol.

Gene	Ratio	Annotation
**Up-regulated genes**		
A1S_2098	12.6	Ethanol dehydrogenase
A1S_2102	13.2	Aldehyde dehydrogenase
A1S_0481	4.4	Phosphate acetyltransferase
A1S_1354	3.7	Azoreductase (flavodoxin_1 superfamily)
A1S_1711	3.3	Homoserine dehydrogenase
A1S_0942	3.5	Nicotinamide mononucleotide transporter
A1S_2664	2	GroEL
A1S_2755	3	Acyltransferase superfamily
A1S_3542	3.2	Hypothetical protein^1^
A1S_2994	2.4	Hypothetical protein
A1S_0913	3.4	Hypothetical protein^2^
A1S_0293	2.4	Hypothetical protein. DUF 1311 superfamily
A1S_1750	3.1	Multidrug efflux protein (AdeB)^3^
A1S_2944	2.6	Hypothetical protein
A1S_3449	2.5	Phosphoenolpyruvate carboxylase
A1S_1698	17	Lipoyl synthase
A1S_0410	2.3	Hca transcriptional activator
A1S_3548	2.3	Hypothetical protein^1^
A1S_0294	2.4	HSP90
A1S_2974	2	Hypoxanthine phosphoriboyltransferase
A1S_3503	2	Hypothetical protein
A1S_2509	2	Putative chaperone^4^
A1S_2506	2.6	Putative diguanylate cyclase
A1S_2325	2.8	Outer membrane protein^5^
A1S_2499	2.6	Hypothetical protein
A1S_2395	3.4	Hypothetical protein, COG1741 Pirin-related protein
A1S_3005	4.7	COG4770 Acetyl-Co carboxylase, alpha-sub.
A1S_2120	2.1	Pseudouridine synthase
A1S_1642	2.6	Putative acyl-CoA dehydrogenase
A1S_1950	2.2	Universal stress protein
A1S_3300	4.6	Acetate permease
A1S_3362	3	Hypothetical protein. Haloacid dehalogenase-like superfamily
A1S_2677	4.5	Phosphodiesterase/alkaline phosphatase D, COG3540
A1S_0970	3.8	Transketolase, C-terminal subunit, COG3958
A1S_1291	2.3	Hypothetical protein, pfam03781:DUF323
A1S_0043	2.5	Phospholipase C
A1S_0997	2.4	Predicted esterase, COG3150
A1S_2020	2.2	RNA binding protein, HicA family
A1S_3543	2.2	Hypothetical protein^1^
A1S_2195	2.6	Hypothetical protein^6^
A1S_3024	2.4	Hypothetical protein
A1S_3231	4.3	Acetyl-CoA hydrolase/transferase domain COG0427
A1S_1641	2.2	Fatty acid desaturase pfam00487
A1S_0115	2.4	Non-ribosomal peptide synthetase,
A1S_3597	4.5	Hypothetical protein^7^
A1S_1727	2.7	LysR transcriptional regulator
A1S_1752	7.5	AdeA multidrug efflux protein
A1S_3418	4.5	4-hydroxyphenylpyruvate dioxygenase, COG3185
A1S_1031	2.1	Protease La
**Repressed Genes**		
A1S_0520	0.46	Putative dehydrogenase, COG0644
A1S_0598	0.42	Hypothetical protein, DUF1768
A1S_0661	0.5	Phage integrase family protein, P-4 like integrase
A1S_0669	0.44	Putative arsenite efflux permease, COG0798
A1S_0923	0.51	Malate:quinone oxidoreductase
A1S_1174	0.32	DNA polymerase V, UmuD subunit
A1S_1266	0.33	Putative transporter protein (Mn2+/Fe2+), COG, 1914
A1S_1267	0.33	Putative lactam utilization protein, LamB/YscF superfamily
A1S_1268	0.31	Hypothetical protein, DUF1445
A1S_1269	0.4	Putative allophanate hydrolase subunit 1 and 2, COG2049/1984
A1S_1270	0.27	Putative carboxylase, COG4770
A1S_1320	0.3	MerR family of transcriptional regulators; highly similar to SoxR
A1S_1334	0.42	Putative L-serine deaminase, COG1760
A1S_1339	0.34	Phenylacetic degradation protein, COG2151
A1S_1398	0.22	Putative ABC His/Gln permease cd03262
A1S_1477	0.37	Predicted branched-chain amino acid permease, COG1296
A1S_2057	0.48	Major Facilitator Superfamily, pfam 07690
A1S_2155	0.32	Putative glutamine amidotransferase, cd03141
A1S_2305	0.22	Putative cation/multidrug pump efflux, COG0841
A1S_2578	0.33	putative non-ribosomal peptide synthetase, pfam08415
A1S_3253	0.58	Hypothetical protein

^1^Only present in Ab ATCC17978, inside pathogenic island 4.

^2^Only predicted to be present in Ab ATCC 17978, next best hit A51_C0660 from *Vibrio cholerae* MZO-3 (62% similarity).

^3^Experimental evidence suggests that AdeB conform an operon with A1S_1752 included in Table 2. In contrast to the rest of the Ab strains, the sequence of ATCC17978 does not have the third gene of the operon.

^4^Only present in Ab ATCC17978 and Acinetobacter sp. ADP1, not identified in Ab ACICU, AYE, and SDF strains.

^5^It shows a truncated domain of the surface antigen superfamily.

^6^Only present in species from the *Acinetobacter* genus.

^7^Only identified in Ab ATCC 17978. This gene is 72% similar to A1S_3595.

We also extracted total RNA from cultures grown to stationary phase in the presence of ethanol and performed a more limited RNA-Seq experiment that produced 146,170 unique reads. The NRR for each gene was calculated and arranged by rank order (Table S2). RNA from stationary phase cells with ethanol exhibited a large number of reads for genes related with the synthesis of siderophores and iron uptake. In fact, in this sample, 33 genes related with iron acquisition are among the 10% of the genes that showed the highest NRR values (Table S2). Interestingly, a putative operon encoding four proteins similar to the high-affinity phosphate transport system were also found among the 10% of the genes with highest NRR. Given the relevance of Fe and phosphate acquisition to bacterial pathogenesis we further explored the expression of these genes by qRT-PCR or using *lacZ* as reporter gene (see below).

### Metabolic effect of ethanol

**Figure 2:**
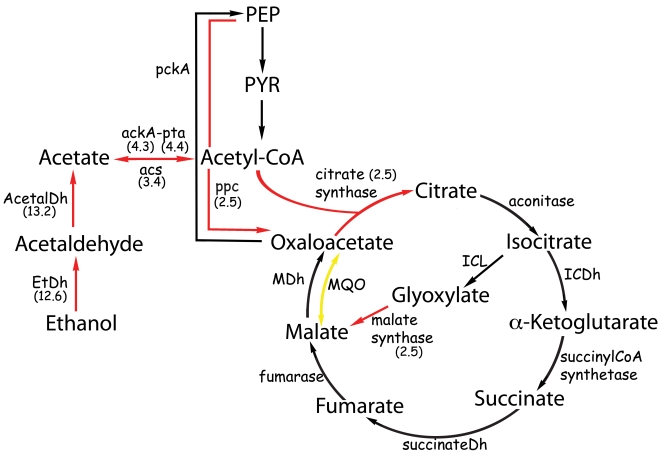
Metabolic pathways affected by the presence of ethanol. The numbers in parenthesis represent the ratio of the number of gene-specific mapped reads from the libraries obtained from cultures grown in ethanol and in the absence of ethanol. EtDh, ethanol dehydrogenase; acetalDh acetaldehyde dehydrogenase; ackA, acetate kinase; pta, phosphate transacetylase; acs, acetyl-CoA synthetase; ICDh, isocitrate dehydrogenase; ICL, isocitrate lyase; MDh, malate dehydrogenase; MQO, malate:quinone oxidoreductase; pckA, phosphoenolpyruvate carboxykinase; ppc, phosphoenolpyruvate carboxylase. Red and yellow arrows represent induced or repressed genes, respectively.

We examined in detail the genes that were induced by growth in the presence of ethanol during exponential phase. The most strongly induced genes encode proteins related to central metabolism or with ethanol/acetate assimilation. These included ethanol dehydrogenase (A1S_2098) and aldehyde dehydrogenase (A1S_2102), which showed an average induction of 12.6 and 13.2-fold, respectively. *A. baumannii* has two other genes that potentially encode a Fe-dependent ethanol dehydrogenase, i.e., A1S_2053, and A1S_2702; these genes are transcriptionally active since they showed a NRR of 28 and 194. However, their expression is not induced by ethanol. Other genes that may encode additional ethanol dehydrogenases are A1S_1788 and A1S_3436. We observed a slight induction of A1S_1788 by ethanol, but it showed a p-value higher than the selected cutoff (Table 2). Therefore, under our experimental conditions, *A. baumannii* seems to be oxidizing ethanol to acetate by the activity of the enzymes encoded by A1S_2098, A1S_2102 and perhaps A1S_1788 (Fig. 2).

**Table 2:** Genes with a P-value above the threshold but showing an induction of two fold or more in two independent experiments.

Gene	fold	P-value	Annotation
A1S_1788	5.3	0.071	Ethanol dehydrogenase Zn-dependent
A1S_0482	4.3	0.084	Acetate kinase
A1S_0704	2.3	0.095	Hypothetical protein pfam 09981: DUF2218
A1S_2148	3.4	0.105	Acetyl-CoA synthetase, COG0365
A1S_1577	23	0.066	flavoprotein involved in K+ transport COG2072
A1S_3301	4.1	0.107	Predicted membrane protein, COG31622.1
A1S_2710	2.5	0.076	Type II Citrate synthase(gltA)
A1S_0179	3.1	0.121	Predicted flavodoxin, COG0655
A1S_3008	4.8	0.115	acetyl-CoA carboxylase, COG4799
A1S_0359	3.9	0.108	Predicted β-lactamase, COG1680
A1S_0618	2.5	0.126	MarR transcriptional regulator, COG1846
A1S_1601	2.7	0.119	Malate synthase G, cd00728
A1S_1380	3.6	0.162	Predicted porine, cd0342
A1S_3167	2	0.186	PilY, COG3419
A1S_3413	3.5	0.131	Amino acid permease cl00524
A1S_3901	2.9	0.084	Hypothetical protein
A1S_1305	3.3	0.065	Outer membrane protein OmpA superfamily
A1S_0355	3.1	0.096	ExonucleaseV gamma subunit

^2.1^This gene is located upstream of A1S_3300, acetate permease, that is included in Table 1. The coding region of these genes is only 1 bp apart, therefore, they may be part of a single operon. The order of these genes is highly conserved in proteobacteria.

In many bacteria, after conversion to acetate, ethanol is further metabolized into acetyl-CoA and then assimilated through the glyoxylate cycle [34],[35]. Acetyl-CoA synthesis occurs by the action of *ackA* and *pta* (acetate kinase and phosphate acetyltransferase, respectively) as has been demonstrated for *Corynebacterium glutamicum* [36],[37] or through *acs* (acetyl-CoA synthetase) as has been demonstrated for *Escherichia coli* [38],[39]. In these bacteria, *ackA* and *pta* are arranged in a bicistronic operon [40],[41]. In *A. baumannii*, these genes are contiguous in the chromosome and therefore may be part of a single operon. From our data, we found that the genes coding for *pta* (A1S_0481) and *ackA* (A1S_0482) showed a 4.4 (Table 1) and 5.3 (Table 2) fold induction in the presence of ethanol. The same situation was found for the gene A1S_2148, which encodes *acs* which was induced 3.4 fold. Both *acs* and *ackA* showed a p-value higher than the threshold (Table 2), but nonetheless, evidence obtained by quantitative RT-PCR corroborated the induction of these two genes by ethanol (see below).

Interestingly, A1S_3300, which encodes an acetate permease, was induced 4.6 fold (Table 1). In *E. coli*, acetate is excreted into the culture medium during exponential phase when the cells are grown in the presence of a high concentration of acetogenic sugars. However, when the culture begins the transition into stationary phase, acetate assimilation begins. In exponential phase, pyruvate is converted to acetate through the action of *ackA* and *pta*, whereas in stationary phase, acetate is assimilated by *acs* [38]. This does not seem to be the case in *A. baumannii*, since our results indicate that during exponential phase all these genes are simultaneously expressed. Therefore, a balance between assimilation and excretion may be occurring.

In many organisms, acetate is metabolized through the glyoxylate cycle, which in conjunction with other reactions of the citric acid cycle (TCA) allows the net synthesis of succinate from two molecules of acetyl-CoA. At the first step of this cycle, acetyl-CoA and oxaloacetate are used to form citrate. Isocitrate is then converted to glyoxylate and succinate, and finally malate is formed from glyoxylate and acetyl-CoA (Fig. 2). The enzymes that carry out these reactions are citrate synthase, aconitase, isocitrate lyase, and malate synthase [34],[35],[42],[43]. We observed that the genes encoding citrate synthase (A1S_2710), and malate synthase G (A1S_1601) were induced 2.5 and 2.7 fold respectively but with a p-value above the threshold (Table 2). qRT-PCR experiments verified that both genes are indeed induced by ethanol (see below). Therefore, acetate assimilation appears to take place through the glyoxylate cycle in *A. baumannii*. In *E. coli* there are two different malate synthases (i.e., malate synthase A and G encoded by *aceB* and *glcB*, respectively) [44]–[46]. This latter gene, *glcB*, codes for a secondary malate synthase that can replace the malate synthase A when *aceB* is mutated [44]. In contrast, only the malate synthase G is present in *A. baumannii*, similar to the situation found in *C. glutamicum* [37].

Unexpectedly, we did not observe an induction of the gene encoding isocitrate lyase (A1S_1008). This may be due to the presence of glucose in the media we used in these experiments, which may have repressed the expression of the isocitrate lyase gene. This result suggests that under these conditions, control of the isocitrate dehydrogenase by phosphorylation is sufficient for activation of the glyoxylate cycle. In *A. baumannii* the genes encoding isocitrate lyase, malate synthase and isocitrate kinase are not encoded by the same operon. Therefore, it is conceivable that these genes are differentially regulated.

We also detected the induction (2.5 fold) of A1S_3449, a gene encoding a putative phosphoenolpyruvate carboxylase (PEPCx) (Table 1). In other bacteria, this enzyme converts phosphoenolpyruvate to oxaloacetate accomplishing an anaplerotic function (Fig. 2) [47]. Another ethanol-induced enzyme related to the metabolism of acetate is a putative acetyl-CoA hydrolase/transferase, encoded by A1S_3231. The product of this gene is highly similar to proteins found in other bacteria, and it is 62% similar to Ach1 (acetyl-CoA hydrolase) from *Saccharomyces cerevisiae*. In *S. cerevisiae*, it has been shown that *ach1* is not involved in the hydrolysis of acetyl-CoA, as originally thought. Instead, Ach1 transfers CoASH from succinyl-CoA to acetate, and this activity is required to support growth in acetate [48]. Due to the high similarity between CoA tranferases and hydrolases the actual activity of A1S_3231 in *A. baumannii* remains to be determined.

We detected a two-fold repression of A1S_3025 which encodes a putative malate∶quinone reductase (MQO). *A. baumannii*, like many other bacteria, possesses two genes that encode for a malate dehydrogenase, a membrane-associated malate∶quinone oxidoreductase (MQO) (A1S_0923), and a cytoplasmic malate dehydrogenase (MDH) (A1S_3025). Our results indicate that the expression level of A1S_3025 did not change in response to ethanol whereas the putative MQO A1S_0923 was down regulated by 50%. Given that MQO is repressed, MDH should be the main enzyme responsible for malate oxidation in this condition (Fig. 2). Similarly, MQO does not seem to play a significant role in malate oxidation in *E. coli* [49], but for *C. glutamicum* it has been shown that the malate∶quinone reductase (MQO) is the main enzyme catalyzing the oxidation of malate to oxalacetate [50]. A 2-fold reduction was also observed for A1S_1334 encoding L-serine deaminase. The reaction catalyzed by this enzyme yields pyruvate and ammonia. It is possible that this reduction helps prevent a further increase in the availability of acetyl-CoA above the level that ethanol catabolism produces.

Overall these results indicate that a variety of metabolic genes are affected by the presence of ethanol and show for the first time the metabolic pathways involved in ethanol assimilation in this bacterium.

### Ethanol induces genes involved in the stress response and pathogenesis

In exponential phase, ethanol elicits the induction of 11 genes that encode hypothetical proteins that do not belong to any pfam or COG already described (Table 1). Five of these genes are unique to *A. baumannii* ATCC17978, and three of them are located in pathogenicity island 4 [20]. Of the remaining six, A1S_2195 is only present in organisms that belong to the *Acinetobacter* genus, and the other five have homologues in many other bacteria (Table 1). Of particular interest, A1S_2509 is present only in *A. baumannii* ATCC17978 and the non-pathogenic *A. baylyi* ADP1. The proteins encoded by these organisms are 40% identical; the first 50 residues of this protein showed high similarity with DjlC (DnaJ- containing domain protein) from *E. coli* and its homologues in other bacteria [28]. Interestingly, A1S_2509 is adjacent to a gene encoding an HSP70-like protein, and this arrangement is conserved among several bacteria [51]. It has been shown that DjlC produces a 10-fold activation of the ATPase activity of the HSP70-like protein [52], and it was proposed that DjlC and HSP70-like were required to respond to certain stress conditions. Therefore, it is possible that A1S_2509 may help to resist the ethanol stress together with A1S_2510 (HSP70-like). Consistent with this hypothesis, we observed that A1S_2510 (HSP70-like) is mildly induced by ethanol (1.4 fold, p-value 0.006) (data not shown).

The A1S_1641 gene, which encodes a fatty acid desaturase, was induced 2.2 fold (Table 1). In other microorganisms, it has been shown that an increase in the amount of unsaturated fatty acids facilitates adaptation to stressful conditions such as acid pH, ionic stress and ethanol [53]–[56]. Other genes induced by ethanol are A1S_1750 and A1S_1752, that likely are part of an operon and encode for an RND-type efflux pump that confers resistance to various antibiotics in *A. baumannii* BM4454 [57]. Interestingly, it has been shown that an RND efflux pump contributes to drug resistance and virulence of *Francisella tularensis* in mice [58]. We also observed the induction of A1S_1950, which encodes a protein that belongs to the universal stress protein family. It is known that members of this family are induced when the cell is exposed to agents that induce stress [59]–[61]. Interestingly, *A. baumannii* has five proteins that belong to this family A1S_1950, A1S_2692, A1S_2072, A1S_0214 and A1S_1246, but only A1S_1950 was induced by ethanol.

Recently, it was shown that overexpression of *rpoH* in *E. coli*, induces a set of genes that were not originally considered as a part of the heat-shock response (HSR); *acpD* is one of these genes [62]. The physiological role of AcpD is still uncertain since its original assignment was as an ACP-phosphohydrolase. AcpD was subsequently shown to be an azoreductase [63]. The induction of *acpD* (A1S_1354) by ethanol supports the idea that this gene is part of a stress response. A reduction of two-fold was detected for a cluster of five genes that could form an operon from A1S_1266 to A1S_1270; unfortunately, the function of these genes is unknown.

One of the aims of this work was to identify potential virulence factors whose expression is induced in presence of ethanol. In this regard, two general traits were observed. First, there was the mild induction of Hsp90, GroEL, and Lon. In many bacterial species, these genes are part of the heat-shock stress response (HSR) [64]–[69]. Second, several genes known to be important for survival under diverse stress conditions exhibited increased expression (Table 1). Members of the HSR are chaperones that refold or prevent aggregation of misfolded proteins [27],[70], and Lon is a protease that hydrolyzes proteins with unstructured regions [71]. In many bacteria, the control of the HSR is mediated by the sigma factor σ^32^, encoded by the *rpoH* gene [27]. It has been shown that the HSR is required for full virulence in some pathogenic bacteria. For instance, DnaJ-like (HSP40) from *Vibrio tapetis* is required for cytotoxicity of hemocytes [28], σ^32^ is required for the invasion of epithelial cells by *Neisseria gonorrhoeae* [29] and the chaperons HSP90 and GroEL of many pathogenic bacteria induce the production of interleukin-8, modulating the immune response [30],[31],[72]. Consistent with these reports, a strain of *A. baumannii* carrying a transposon insertion in the gene encoding for σ^32^ (*rpoH*) was shown previously to be avirulent in the presence of ethanol towards *C. elegans* and *D. discoideum* [20]. Therefore, it is conceivable that ethanol could exacerbate the virulence of *A. baumannii* through the induction of heat-shock proteins, such as Hsp90, GroEL and Lon. The products of other genes listed in Table 1 may also help *A. baumannii* to tolerate stress conditions. Since it has been shown that one stress response might help bacteria to contend with other stress conditions [73]–[76], it is attractive to hypothesize that ethanol could improve the ability of *A. baumannii* to survive in the host since that several pathways of stress responses are activated.

Among the genes that we detected as induced by ethanol (Table 1), A1S_0043 encoding a phospholipase C deserves particular attention. This protein has been recognized as a virulence factor in other pathogenic bacteria [77]–[79]. For this reason, we further analyzed this gene as described below.

### Quantitative RT-PCR of selected targets

To validate the induction of some of the genes identified by RNA-Seq (Table 1), we carried out qRT-PCR experiments. The expression of A1S_2664, A1S_0294, and A1S_0043 that encode for GroEL, HSP90 and phospholipase C, respectively, was measured, along with several genes whose expression was induced by ethanol but showed a p-value above the threshold (Table 2). These latter genes were A1S_0482, A1S_2148, A1S_2710, and A1S_1601 which encode acetate kinase, acetyl_CoA synthetase, citrate synthase, and malate dehydrogenase G, respectively. Ethanol dehydrogenase (A1S_2098) was used as a positive control. The down-regulated gene A1S_1266 was also included in this analysis. A1S_2846 and A1S_0880 were used as internal controls to calculate the fold-change after treatment with ethanol (see methods section). To test the expression of the genes above mentioned, we used total RNA isolated from exponential cultures of *A. baumannii* grown in the absence or presence of ethanol.

**Figure 3:**
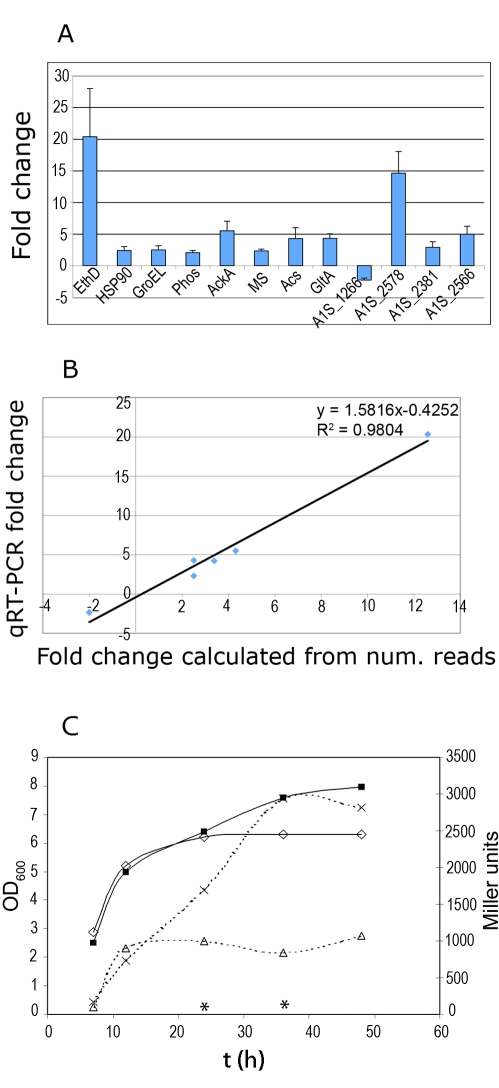
Fold change of selected genes determined by qRT-PCR. (A) Fold change of the expression levels of genes A1S_2098 encoding ethanol dehydrogenase (fold change 20.3); A1S_0294, HSP90 (2.38); A1S_2664, GroEL (2.46); A1S_0043, phospholipase C (2.02); A1S_0482, acetate kinase (5.5); A1S_1601, malate synthase G (2.3); A1S_2148 acetyl-CoA synthetase (4.25); A1S_2710, citrate synthase (4.3); A1S_1266, hypothetical protein (−2.3). These genes were tested using RNA obtained from cultures grown in 1.1% ethanol and without ethanol. The genes tested using RNA obtained from stationary phase cultures with and without ethanol are: A1S_2578, A1S_2381, and A1S_2566, encoding a putative non-ribosomal peptide synthetase (similar to the subunit F of the enterobactin synthetase), acinetobactin synthetase subunit E, and a siderophore receptor protein, respectively. (B) Correlation between the expression ratios of selected genes determined by qRT-PCR and RNA-Seq. (C) Time course of expression of the *ptsS-lacZ* fusion along the growth curve. *A. baumannii* cells carrying the plasmid expressing *pstS*-*lacZ* were grown in YPDA medium in the presence or absence of ethanol. At the indicated times, cell growth was monitored as culture turbidity at 600nm (continuous lines) from cultures grown in the absence (open squares) or presence (filled squares) of ethanol. β-Galactosidase activity (dashed lines) was determined from cultures grown in the absence (open triangles) or presence (multiplication symbol) of ethanol. Asterisks indicate the β-galactosidase activity from cultures grown in the presence of 10 mM phosphate buffer. These time points showed an OD_600_ of 6 and 6.2, respectively. All results are the mean of three experiments, with standard deviations of less than 15%.

As shown in Fig. 3A, qRT-PCR experiments confirmed that the expression of all these genes was regulated by ethanol. Moreover, the fold change detected for each gene was similar to the ratio of induction and repression observed by RNA-Seq (Fig. 3B). These results support the conclusions outlined in the previous section regarding ethanol metabolism.

To validate the high expression level of the genes detected in the samples obtained from stationary phase cultures grown in the presence of ethanol, we also measured the expression levels of A1S_2381, A1S_2566, and A1S_2578 by qRT-PCR. These genes were randomly chosen among the genes that are related to Fe uptake and showed a high number of reads (NRR) (Table S2). A1S_2381 is required for acinetobactin synthesis and is located within a locus of 13 genes that are involved in the synthesis and transport of acinetobactin in *A. baumannii* ATCC19606 and *A. baumannii* ATCC17978. In contrast, A1S_2566 and A1S_2578 (encoding a protein required for siderophore synthesis and a siderophore receptor, respectively) are part of a second locus for Fe acquisition that is present in *A. baumannii* ATCC17978 but not in *A. baumannii* ATCC19606 [17]–[19]. To test the expression of these genes, we used total RNA from stationary phase cells that were grown in the presence or absence of ethanol (see methods). As shown in Fig. 3B, the expression of these genes was induced by the presence of ethanol in the culture medium, although this effect could be indirect given that a higher O.D._600_ is reached when ethanol is included in the culture medium [24] (see panel C in Figure 3).

We also tested the induction of expression for the genes that belong to the putative *pts* operon (A1S_2448 to A1S_2445) which encode a high-affinity phosphate transport system. For this study, we used *lacZ* as reporter gene. The promoter region upstream of A1S_2448 (homologous to *pstS*) was cloned into pMP220. The amount of β-galactosidase produced by *A. baumannii* transformed with this construction was low when 10 mM PO_4_ was included in the culture medium (Fig. 3C, measures indicated by asterisks). The expression of *lacZ* was evaluated in *A. baumannii* cells grown in the absence or presence of ethanol at different time points of incubation. As shown in Fig. 3C, a low level of β-galactosidase activity was detected for both cultures at OD_600_ <3, but higher activities were observed as the cultures approached stationary phase. At 24 h of incubation, in the presence of ethanol higher amounts of β-galactosidase were detected nevertheless both cultures showed a similar OD indicating that increased β-galactosidase was not due to higher cell numbers (Fig. 3C). To ensure that plasmid integrity was intact through these experiments, we rescued the plasmids from both ethanol treated and untreated cultures at the end of the experiment and found that in each case the plasmid were functional and without detectable rearrangements. Thus, these results indicate that the high-affinity phosphate transport system of *A. baumannii* is highly expressed at high cell densities and that A1S_2448 is induced after incubation with ethanol.

The induction of the transport genes A1S_2448-45 is of particular significance since it has been demonstrated previously that a strain of *A. baumannii* carrying an insertion in A1S_2447 (homologue of *ptsC*) was avirulent towards *C. elegans* and *D. discoideum* [20]. Therefore, it is conceivable that ethanol could exacerbate the virulence of *A. baumannii* taking advantage of the induction of these uptake systems (Fe and phosphate) that in other bacteria have also been related with virulence [26].

It has been reported that community-acquired *Acinetobacter* infections are associated with underlying conditions such as alcoholism, smoking, chronic obstructive pulmonary disease and diabetes [80]–[82]. Our results provide mechanistic insight into how ethanol may modulate *A. baumannii* infections. Furthermore, the molecular mechanism underlying this effect could be multifactorial, given that ethanol up regulates TLR2 causing inflammation of the airway epithelium [83]. Ethanol also induces a delay of viability loss in stationary-phase cultures of bacteria [84], and we demonstrate that ethanol induces a stress response that may give the pathogen a better fitness to survive in the host.

### Generation of a plc1 mutant strain

One of the genes that was induced by ethanol encoded a phospholipase C (plc; A1S_0043). *A. baumannii* has another gene encoding a phospholipase C (A1S_2055). Both genes are absent in the non-pathogenic *A. baylyi* ADP1 but present in other strains of *A. baumannii* that have been sequenced [23]. The proteins encoded by A1S_0043 and A1S_2055, show a similarity of 72%, and both are highly similar (75% similarity) to the phospholipases reported for *Burkholderia pseudomallei*. As is the case for phospholipase C proteins in other bacteria [85],[86], the N-terminal region contains the conserved residues that are recognized by the twin-arginine secretion system; therefore both phospholipases may be secreted. In addition, from the gene arrangement it can be proposed that A1S_0043 is expressed as a monocistronic mRNA since no other coding region is predicted to be located in the adjacent 404 bp downstream A1S_0043, and the upstream ORF, A1S_3479, is transcribed in opposite direction.

To evaluate the contribution of the phospholipase C (encoded by A1S_0043, from here on referred to as *plc1*) to *A. baumannii* virulence, we isolated a mutant strain carrying the insertion of a kanamycin cassette in the coding region of *plc1* (see methods). This mutant strain did not show any apparent growth defects upon growth in liquid medium (data not shown). The ability of this strain to produce cellular damage on a monolayer of epithelial cells was tested as described below.

### Infection of epithelial cells

**Figure 4:**
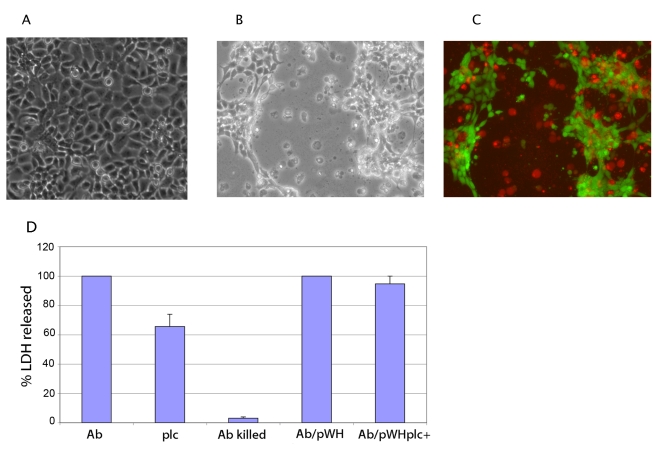
Cell damage associated with *A. baumannii* infection. (A) Uninfected monolayer of FaDu cells observed at 150X with phase-contrast microscopy; (B) FaDu cells infected with *A. baumannii* after 18 h of incubation and observed at 150X with phase-contrast microscopy; (C) the same monolayer shown in panel B, stained with LIVE/DEAD reagent (Invitrogen) and observed by fluorescence microscopy. The live cells show green fluorescence whereas dead cells are observed as red. (D) Percentage of LDH released to the culture medium after 22 h of infection with *A. baumannii* wild-type, (Ab); *plc1Δ*::kan mutant, (plc); *A. baumannii* wild-type carrying pWH1266-Gm (Ab/pWH); *plc1Δ*::kan mutant carrying pWH1266-Gm/*plc1*+ (plc/pWHplc^+^); *A. baumannii* cells killed with formaldehyde, (Ab killed).

Incubating a monolayer of epithelial cells in the presence of *A. baumannii* has been reported to elicit several morphological and physiological changes, such as loss of viability as revealed by trypan blue staining, detachment from the culture plate, and a general shrinking of the cells [14],[87]. Consistent with these reports we found that after infection, FaDu epithelial cells became permeable to trypan blue indicating that *A. baumannii* compromises the membrane permeability. Furthermore, after 18 h of infection we observed extensive detachment of the cell monolayer and cellular death in many of the remaining cells (Fig. 4B and C; stained non-infected controls are shown in Fig. S1). It has been shown that the intracellular enzyme lactate dehydrogenase (LDH) is released into the culture medium after any insult that compromises the integrity of the plasma membrane. Therefore, we assessed cell damage by measuring LDH release upon infection with *A. baumannii*. For this assay, a monolayer of FaDu cells was infected with *A. baumannii* and the amount of LDH released was measured after 22 h of incubation. The amount of damage produced by the different strains was estimated as percent of the amount LDH released when the cells were infected with wild-type *A. baumannii*. As shown in Fig. 4D, only live bacteria triggered LDH release indicating that cell damage is a consequence of the bacterial infection.

Using this assay, the cytotoxic effect produced by the strain carrying the *plc1Δ*::kan allele was determined. A reduction in the amount of LDH released into the culture medium upon infection with this strain was observed (Fig. 4D). To further validate this result an infection was carried out with either the wild-type or the *plc1* mutant transformed with pWH1266-Gm (empty vector) and pWH1266-Gm containing the *plc1* gene, respectively. Cells infected with wild-type and *plc* mutant strain containing the *plc1*+ gene released a similar amount of LDH (Fig. 4D), indicating that phospholipase C contributes to cause cellular damage. Bacteria recovered from the infection plate were used to confirm that the complementing plasmid was stably maintained without detectable rearrangements (data not shown).

Phospholipase C has been reported as a virulence factor in many bacteria, such as *Pseudomonas aeruginosa*, *Legionella monocytogenes* and in the Gram-positive bacteria *Clostridium perfringens* [77],[88]. *P. aeruginosa* has an acidic phospholipase that shows strong hemolytic activity, and contributes to its ability to cause cellular damage [89]. Recently it has been shown that *L. monocytogenes* uses a phosphatidylinositol-specific phospholipase C to escape efficiently from the phagosome in macrophages [79],[90], and it also has a phosphatidylcholine-prefering phospholipase C that is involved in the escape from the phagosome in epithelial cells [91],[92]. *B. pseudomallei* has two phospholipases C that hydrolyze phosphatidylcholine and sphingomyelin and neither is hemolytic for human erythrocytes. However, it was reported that Plc-2 has a significant role in the virulence of this pathogen towards HeLa cells, whereas Plc-1 seems to have a minor one [78]. Thus, our results demonstrating that *A. baumannii* phospholipase C is important for virulence is consistent with finding with several other organisms.

Overall our results demonstrate a number of important conclusions:

First, RNA-Seq provides a comprehensive, detailed overview of the bacterial transcriptome. Even though contaminating rDNA reads can be present in large numbers, large numbers of unique reads can still be obtained by deep sequencing.

Second, in *A. baumannii*, ethanol is efficiently assimilated as a carbon source through the glyoxylate cycle that is a pathway required for full virulence in many pathogens.

Third, ethanol induces the expression of many proteins related to stress, including UspA, Hsp90, GroEL, Lon. Specific stress responses may help bacteria to contend with adverse condition pathogens face during infection.

Fourth, ethanol induces the expression of a phospholipase C that contributes to *A. baumannii* cytotoxicity.

Fifth, ethanol promotes the growth of *A. baumannii*, and presumably during stationary phase the resources of the medium are more efficiently utilized by the induction of the two Fe uptake systems and the high-affinity phosphate transporter system. Thus, overall these studies contribute a wealth of new information into the pathogenic response of *Acinetobacter baumannii*.

## Methods

### Bacterial strains, plasmids, oligonucleotides, and growth conditions

*A. baumannii* ATCC17978 was grown in YPDA culture medium (1% yeast extract, 2% peptone, 2% dextrose, and 0.012% adenine sulfate) at room temperature, or at the temperature indicated. When indicated, 1.1% ethanol was added to the culture medium. In this study 1.1% ethanol was used instead of 1% ethanol as previously published [20] because 1.1% ethanol caused more reproducible effects previous reported on *A. baumannii* growth. Bacterial strains were also grown in LB. When required, antibiotics were added at the following concentrations: ampicillin (100 µg/ml), kanamycin (50 µg/ml), gentamicin (20 µg/ml), tetracycline (10 µg/ml). Cloning were performed using the plasmids pCR2.1-TOPO (Invitrogen) and pUC19R (Invitrogen). pUC4K was used as source of the kanamycin cassette (GE Healtcare Life Sciences). pJQ200 was used as suicide plasmid [93]. pJQ200 confers Gm^R^ and carries the *sacB* gene from *Bacillus subtilis* to counterselect the presence of the plasmid in the presence of sucrose. This plasmid was also used as a source of the Gm^R^ cassette. pWH1266 was used as a shuttle vector for *E. coli* and *A. baumannii* [94]. pMP220 was used to construct transcriptional fusions to the promoter-less *lacZ* [95]. The sequences of the oligonucleotides used in this work are in Table S3.

### Recombinant DNA techniques

Chromosomal DNA was obtained using the GenElute Bacterial Genomic DNA kit from Sigma-Aldrich. Plasmids were purified using the plasmid purification kit from Qiagen. DNA was amplified using PrimeStar HS Taq polymerase or LA Taq polymerase (Takara) according to the recommendations of the manufacturer. Transformation of *E. coli* was carried out using CaCl_2_ competent cells [96]. Electroporation was used to transform *A. baumannii* cells, following the protocols previously reported [97].

### RNA isolation

*A baumannii* cultures were grown to mid-log phase (OD_600 nm_ = 1.8) in YPDA or YPDA with 1.1% ethanol, at room temperature and shaking of 150 rpm. The cells were collected at 4°C and the RNA was isolated using the RiboPure-Bacteria kit (Ambion) according to the manufacturer's instructions. Residual DNA in the samples was removed using DNaseI. The integrity of the RNA was analyzed using an Agilent bioanalyzer (Agilent technologies). The MICROBExpress kit (Ambion) was used to remove the 23S and 16S rRNA from the total RNA samples. When indicated, the samples of enriched mRNA were subject to a final step of purification using the Megaclear kit from Ambion. To evaluate the degree of rRNA depletion the samples were analyzed on an Agilent bioanalyzer. The same protocol was used to isolate RNA from *A. baumannii* cultures from stationary phase. In this case, the cells were collected three hours after the OD_600_ of the cultures did not show any further increase.

### cDNA synthesis and preparation of the library for high-throughput sequencing

The first two libraries (corresponding to the cultures with or without ethanol) were obtained using 2 µg of enriched mRNA that was depleted only once with the ribo-minus beads included in the MICROBExpress kit. For the samples corresponding to the biological duplicates, we performed two steps of depletion with ribo-minus beads and an additional purification step using the Megaclear kit. Double-stranded cDNA was obtained using hexameric random primers and the Super-script double-stranded cDNA synthesis kit from Invitrogen. The cDNA was purified using the Qiaquick PCR Purification kit from Qiagen, and subject to a partial digestion with DNaseI in order to obtain a substantial enrichment of fragments between 100 and 300 bp. After digestion, the sample was loaded on a 1.2% agarose gel, and the fragments between 100 and 300 bp were purified using the Qiaquick Gel Extraction kit from Qiagen. The ends of the fragments were end-repaired and A-tailed. For this, the End-It kit (Epicenter) was used according to the manufacturer's instructions. The repaired cDNA was purified using the Qiaquick PCR purification kit and A-tailed using the Klenow fragment of the DNase polymerase (NewEngland-BioLabs) and dATP. The sample was purified and ligated to the genomic adapters provided by Illumina. After ligation, the sample was loaded on a 2% agarose E-gel (Invitrogen), and the fragments between 150 and 350 bp were excised from the gel and purified using the Qiaquick gel extraction kit. A PCR reaction of the gel-purified cDNA was performed in 50 µl using the 1X master mix Phusion-High Fidelity DNA polymerase, and the primers 1.1 and 2.1 provided by Ilumina. The reaction was amplified with 17 cycles, and the sample loaded on a 1.2% agarose gel and the fragments between 150 and 350 bp were excised from the gel and purified. The sample was quantified spectrophotometrically using a nanodrop (Thermo) and sequenced in a Genome analyzer II (Illumina).

### Data analysis

The raw reads of 35 bp were truncated as 28-mers and remapped with the Efficient Local Alignment of Nucleotide Data (ELAND) allowing for 1 and 2 nt mismatches. The output file containing only the sequences that mapped once in the genome was further analyzed to ascertain genome coverage and to assign the number of reads per locus (orf or intercistronic region). To identify the genes regulated by ethanol, the libraries were initially compared by pairs; for this, the number of reads for each coding region was determined, the number of total reads was normalized between these libraries and the ratio of reads between ethanol and no ethanol was calculated. The genes that showed a ratio larger than 1.9 and lower that 0.5 were considered potential candidates. Finally, the number of reads for the four libraries was normalized and the Student's *t*-test was applied for each gene. Those genes that showed a P-value lower or equal to 0.05 were considered as genes regulated by ethanol. To obtain information regarding the level of expression among the genes, we calculate the number of relative reads (NRR) per coding region using a window of 250 bp.

### Quantitative RT-PCR

The RNA was isolated as described in the previous section. As templates for this assay we used the same RNA samples that were used for the synthesis of first Illumina libraries and two additional pair of samples that were independently obtained. The reverse transcription step was carried out using the iScript Select cDNA Synthesis Kit from Bio-Rad, according to the manufacturer's instructions. The primer3 software [98] was used to select primers that would amplify a product of approximately 200 bp. The quantitative real-time PCR assay was performed with SYBR-Green I master mix (Applied Biosystems) in a LightCycler 480 system. Reactions were set up according to the manufacturer's instructions, and three technical replicates for each sample were included. The amplification conditions were: 95°C, 5 min (ramp/rate of 4.8°C/s), followed by 45 cycles of 95°C 10 sec (ramp/rate 4.8°C/s), 55°C 20 sec (ramp/rate 2.5°C/s), and 72°C 30 sec (ramp/rate 4.8°C/s). The specificity of the reaction was confirmed by obtaining a melting curve from 95 to 55°C and visualizing the amplified product in a 5% polyacrylamide/TAE gel. The absence of product using only RNA in the PCR reaction (without reverse transcriptase) was also verified. The Cp value was defined as the cycle in which the fluorescence value was above the background. The efficiency of the amplifications for each pair of primers was determined obtaining a standard curve using serial dilutions of DNA. The efficiency was calculated using the formula E = 10(1/-s)X100 where s is the slope of the curve. The fold change was calculated using the 2^−ΔΔCt^ (2^−ΔΔCp^) method [99]. A1S_2846 encoding a putative sulfite reductase was used as internal control. Similar results were obtained if A1S_0880 encoding MinC was used as internal control instead of A1S_2846.

### Isolation of mutant strains and recombinant plasmids

To obtain the *A. baumannii* mutant strain in the *plc1* gene, a fragment of 2579 bp carrying the *plc1* gene was amplified by PCR using the oligonucleotides A1S_0043.1 and A1S_0043.2 and cloned into pCR2.1-TOPO plasmid. An internal fragment of 519 bp from the coding region of *plc1* was removed by inverse PCR using the oligonucleotides A1S_0043.A and A1S_0043.B and substituted with a kanamycin resistance cassette. The DNA fragment carrying the *plc1Δ*::kan allele was subcloned into pJQ200. The resultant plasmid was used to electroporate *A. baumannii* cells. Single recombinants appeared after overnight incubation on LB plates in the presence of gentamicin. Double recombinants were selected plating serial dilutions of different Gm^R^ colonies on LB plates with kanamycin and 3% sucrose. The proper replacement was confirmed by PCR. Plasmid pWH1266 (Ap^R^ Tc^R^) that is stable in *A. baumannii* was used to carry the *plc1* gene. To generate this construct a PCR fragment carrying *plc1* (oligonucleotides A1S_0043.1 and A1S_0043.2) was cloned into pWH1266 using the BamH1 and SalI sites. It has been shown that a gene cloned in these sites is expressed under control of the Tet promoter. The resulting plasmid does not confer any Tc resistance and given that *A. baumannii* ATCC17978 is Ap^R^, we proceeded to construct the plasmids pWH1266-Kan and pWH1266-Gm, in which a kanamycin or gentamicin resistance cassette was cloned into the EcoRI site of pWH1266. The gene conferring Gm^R^ resistance was obtained by PCR using the oligonucleotides acc3 and acc4. The gene conferring Kan^R^ was obtained by PCR using the oligonucleotides Kanfw1 and Kanrev1. The *plc1* gene was cloned in pWH1266-Kan^R^ and pMH266-Gm^R^. As a control the *gfp* gene was cloned in pWH1266-Kan and introduced to *A. baumannii*; as expected, green-fluorescent cells were observed. The pMP220/2248p plasmid carrying the transcriptional fusion of *lacZ* to the control region of A1S_2448 was constructed by cloning a PCR fragment of 913 bp obtained by PCR using the oligonucleotides AB2448up2 and AB2448dw5. This fragment carries the promoter region located upstream of A1S_2448.

### β-Galactosidase assay

*A. baumannii* cells carrying pMP220/2248p were grown in YPDA-Tc, or YPDA-Tc supplemented with 1% ethanol, or 10 mM PO_4_ buffer pH 7. The cultures were grown aerobically at 30°C and aliquots were assay at different time points. β-galactosidase activity was determined in Chloroform/SDS-permeabilised cells. Hydrolysis of *o*-nitrophenyl-β-D-galactopyranoside was carried out at 37°C. Activities are expressed in terms of cell density using the formula of Miller [100].

### Lactate dehydrogenase (LDH) assay

The FaDu cell line originating from a hypopharyngeal carcinoma was obtained from ATCC (ATCC HTB-43). The cell line was grown under 5% CO_2_ at 37°C in Eagle's minimum essential medium with Earle's balanced salt solution (ATCC 30–203) supplemented with 10% heat-inactivated fetal bovine serum (Gibco 16140) and 1% of a solution containing penicillin/streptomycin at 10,000 U/ml and 10 mg/ml, respectively (Gibco 15140). The cells were seeded in 12 well-plates and infected when they reached 5×10^5^–7×10^5^ cells per well. Before infection, the monolayer of epithelial cells was carefully washed with PBS, and fresh medium without antibiotics was added. Bacterial strains were grown on plates of LB or LB with the appropriate antibiotic and incubated overnight at 37°C. The next day a suspension of bacterial cells was prepared in PBS, and the OD_600_ was registered and adjusted (2 OD_600 nm_ = 1×10^9^ cells/ml). Formaldehyde-fixed bacteria were prepared by incubation of the suspension in 1% formaldehyde for 4 h at 4°C as described [101]. The epithelial cells were infected at an MOI of 100 with no more than 10 µl of bacterial suspension. Mock-infections and infections were done in duplicate. The plates were centrifuged at 1,500 rpm for 5 min and then incubated for 22 hrs at 37°C and 5% CO_2_. The amount of LDH released into the culture medium was determined according to the manufacturer's instructions (BioVision Research Products. Mountain View, CA). Each set of experiments was performed in triplicate. Bacteria recovered from the infection plate were used to determine the number of colony forming units on plates with and without gentamicin. The restriction pattern of the plasmid obtained from these cells was analyzed by double digestions with EcoRI and SalI or EcoRI and BamHI.

## Supporting Information

Figure S1 Monolayer of uninfected cells stained with LIVE/DEAD reagent.
(1.36 MB TIF)

Table S1 Genes showing a NRR less than 1.
(0.03 MB DOC)

Table S2 Genes highly expressed in stationary phase in the presence of ethanol.
(0.05 MB XLS)

Table S3 Oligonucleotides used in this work.
(0.02 MB DOC)
