# Supplementary figures and images for: Molecular Mechanisms of Ethanol-Induced Pathogenesis Revealed by RNA-Sequencing
Source: PLoS Pathog. 2010 Apr 1;6(4):e1000834. doi: 10.1371/journal.ppat.1000834 (PMC2848557; doi:10.1371/journal.ppat.1000834)

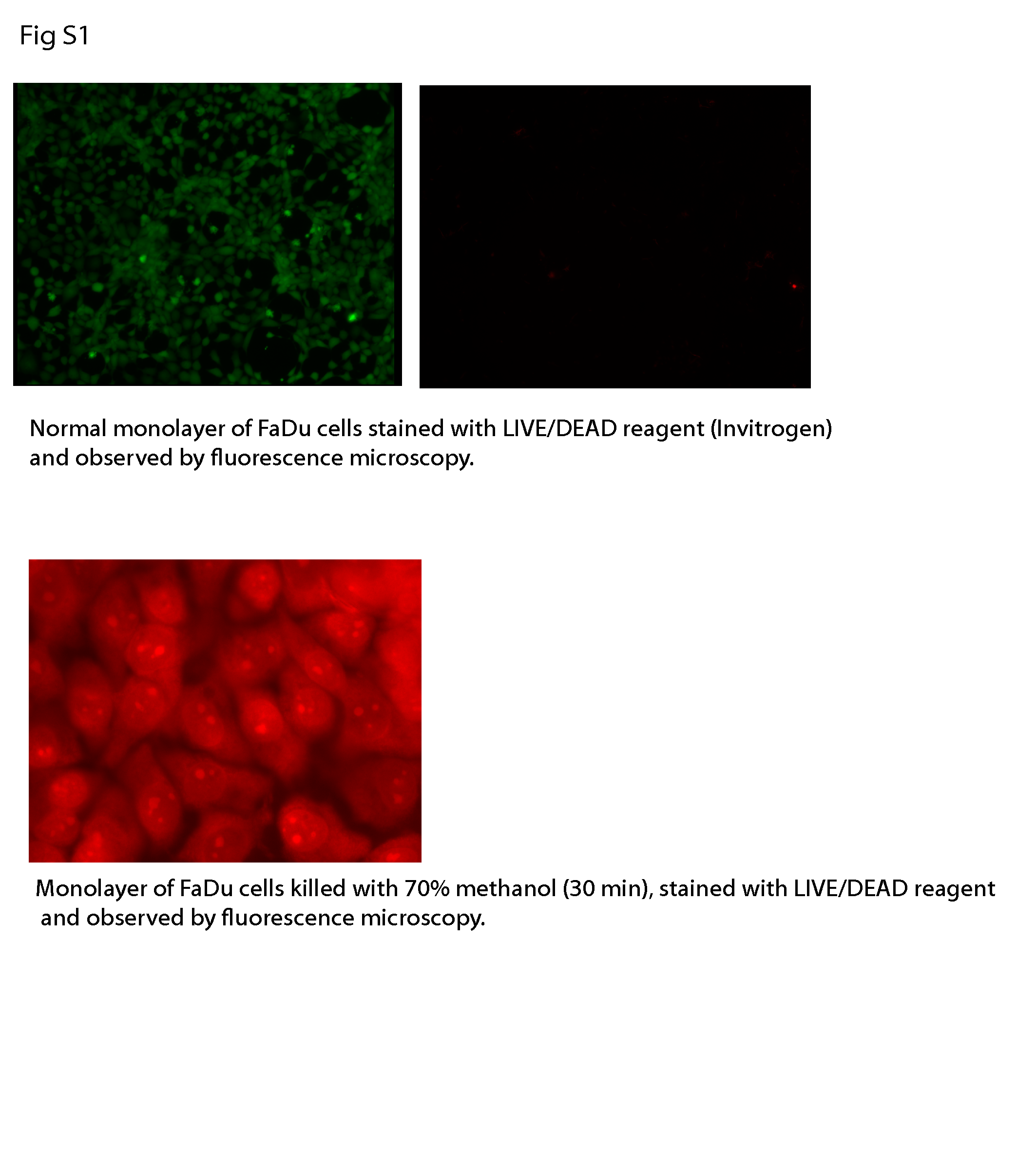

Supplement: Figure S1 — Monolayer of uninfected cells stained with LIVE/DEAD reagent. (1.36 MB TIF) [file ppat.1000834.s001.tif]
