# Supplementary material for: Molecular Mechanisms of Ethanol-Induced Pathogenesis Revealed by RNA-Sequencing
Source: PLoS Pathog. 2010 Apr 1;6(4):e1000834. doi: 10.1371/journal.ppat.1000834 (PMC2848557; doi:10.1371/journal.ppat.1000834)
Supplement: Table S1 — Genes showing a NRR less than 1. (0.03 MB DOC) [file ppat.1000834.s002.doc]

**Table S1. Genes showing a NRR less than 1.**

**______________________________________________________________________**

Gene size (bp) annotated as

______________________________________________________________________

A1S_0109 551 homoserine lactone synthase

A1S_0182 1259 hyp. Prot.

A1S_0464 770 TatC

A1S_0664 881 repC

A1S_0725 1406 tricarballylate dehydogenase

A1S_0727 794 putative periplasmic transport

A1S_0739 551 transcriptional regulator TetR-family

A1S_0741 272 hyp. Prot.

A1S_0743 329 hyp. Prot.

A1S_0919 380 putative permease

A1S_0948 764 fabG

A1S_0950 344 nirB

A1S_0959 1196 hyp. Prot.

A1S_0967 749 fabG reductase

A1S_0969 845 transketolase

A1S_1078 818 esterase/lipase

A1S_1081 551 transcriptional regulator TetR-family

A1S_1106 536 hyp. Prot.

A1S_1107 1250 porin

A1S_1108 1139 acyl-CoA dehydrogenase

A1S_1117 1346 vanillate transporter

A1S_1125 875 putative transferase

A1S_1143 440 hyp. Prot.

A1S_1149 428 hyp. Prot.

A1S_1152 1526 putative helicase

A1S_1165 1133 putative phage tail tape measure protein

A1S_1169 281 hyp. Prot.

A1S_1171 542 putative lysozyme

A1S_1197 1394 putative extracellular nuclease

A1S_1209 1262 putative benzoate transport protein

A1S_1214 500 putative benzoate 12-dioxygenase sub b

A1S_1215 1382 putative benzoate 12-dioxygenase subunit alpha

A1S_1276 662 hyp. Prot.

A1S_1278 632 allophanate hydrolase

A1S_1311 257 hyp. Prot.

A1S_1359 1037 ABC-type Fe3+ transport system

A1S_1360 380 ABC transporter

A1S_1362 1709 ABC-type Fe3+ transport system

A1S_1384 389 CinA competence damage protein

A1S_1394 959 two-components respone regulator

A1S_1422 884 triphosphoribosyl-dephospho-CoA synthase

A1S_1440 1466 msf transporter

A1S_1447 1469 permease

A1S_1459 275 putative alkyl hydroperoxide reductase subunit F

A1S_1494 1316 DcaP-like

A1S_1583 296 hyp. Prot.

A1S_1585 1331 DNA helicase

A1S_1587 524 EsvK2

A1S_1588 1139 Phage terminase-like

A1S_1590 662 peptidase U35 phage prohead HK97

A1S_1596 1097 hyp. Prot.

A1S_1600 548 lysozyme

A1S_1681 767 putative methyltransferase

A1S_1722 839 ABC transporter

A1S_1741 1250 esterase/lipase

A1S_1742 785 oxidoreductase

A1S_1743 1238 putative oxygenase

A1S_1744 530 flavin-reductase

A1S_1746 1058 transcriptional regulator AraC family

A1S_1758 854 short chain oxidoreductase

A1S_1763 551 putative transcriptional regulator

A1S_1768 1160 hyp. Prot.

A1S_1769 1328 RND efflux permease

A1S_1772 1538 msf transporter

A1S_1776 341 putative transcriptional regulator

A1S_1782 803 hyp. Prot.

A1S_1783 788 transcriptional regulator AraC family

A1S_1784 491 GNAT family acetyltransferase

A1S_1796 497 aldehyde dehydrogenase

A1S_1808 1289 putative anion permease

A1S_1813 578 hyp. Prot.

A1S_1837 815 msf transporter

A1S_1838 260 msf transporter

A1S_1847 653 oxoadipate CoA-transferase

A1S_1848 191 Beta-ketoadipyl CoA thiolase

A1S_1853 1277 hyp. Prot.

A1S_1854 2306 Cu2+-containing amine oxidase

A1S_1855 914 Transcriptional regulator FeaR

A1S_1867 1349 msf transporter

A1S_1868 575 porin for benzoate transport

A1S_1892 191 Beta-ketoadipyl CoA thiolase

A1S_1893 653 3-oxoadipate CoA-transferase subunit B

A1S_1956 1406 put aa permease

A1S_2006 590 two-components Response Regulator. ANTAR domain

A1S_2015 1292 polV (UmuC UmuD)

A1S_2016 509 Phage related lysozyme

A1S_2031 470 hyp. Prot.

A1S_2033 428 hyp. Prot.

A1S_2074 425 hyp. Prot.

A1S_2105 323 ethanolamine ammonia-lyase

A1S_2208 554 putative transcriptional regulator

A1S_2333 536 peptidase C39

A1S_2373 755 acinetobactin biosynthesis

A1S_2376 269 multidrug resistance protein

A1S_2381 1628 enterobactin synthase sub E

A1S_2388 947 ferric acinetobactin transport system

A1S_2396 563 transcriptional regulator TetR-family

A1S_2399 899 dihydroflavonol 4-reductase

A1S_2408 293 metal hydrolase

A1S_2410 1019 fatty acid desaturase

A1S_2557 395 hyp. Prot.

A1S_2624 659 CPS-53 prophage bactoprenol glucosyl transferase

A1S_2647 578 putative transcriptional regulator TetR family

A1S_2744 584 SAM-dependent methyltransferase

A1S_2885 380 putative signal peptide protein

A1S_3122 893 hyp. Prot.

A1S_3251 599 transporter LysE

A1S_3255 812 putative transcriptional regulator AraC family

A1S_3264 551 putative transcriptional regulator

A1S_3266 287 aldo/keto reductase

A1S_3291 341 alginate biosynthetic membrane

A1S_3559 107 hyp. Prot.

A1S_3562 101 hyp. Prot.

A1S_3567 98 hyp. Prot.

A1S_3570 101 hyp. Prot.

A1S_3576 284 hyp. Prot.

A1S_3578 95 hyp. Prot.

A1S_3586 128 hyp. Prot.

A1S_3601 110 hyp. Prot.

A1S_3603 407 hyp. Prot.

A1S_3604 251 hyp. Prot.

A1S_3613 215 hyp. Prot.

A1S_3616 299 hyp. Prot.

A1S_3621 389 hyp. Prot.

A1S_3647 116 hyp. Prot.

A1S_3650 83 hyp. Prot.

A1S_3655 101 hyp. Prot.

A1S_3660 260 hyp. Prot.

A1S_3663 107 hyp. Prot.

A1S_3669 101 hyp. Prot.

A1S_3672 128 hyp. Prot.

A1S_3683 251 hyp. Prot.

A1S_3691 122 hyp. Prot.

A1S_3694 530 hyp. Prot.

A1S_3695 182 hyp. Prot.

A1S_3696 263 hyp. Prot.

A1S_3697 482 hyp. Prot.

A1S_3698 194 hyp. Prot.

A1S_3704 308 hyp. Prot.

A1S_3705 362 hyp. Prot.

A1S_3717 104 hyp. Prot.

A1S_3724 98 hyp. Prot.

A1S_3728 98 hyp. Prot.

A1S_3736 116 hyp. Prot.

A1S_3737 170 hyp. Prot.

A1S_3742 125 hyp. Prot.

A1S_3752 107 hyp. Prot.

A1S_3755 275 hyp. Prot.

A1S_3769 410 hyp. Prot.

A1S_3771 950 hyp. Prot.

A1S_3777 251 hyp. Prot.

A1S_3778 407 hyp. Prot.

A1S_3782 173 hyp. Prot.

A1S_3783 233 hyp. Prot.

A1S_3784 470 hyp. Prot.

A1S_3793 185 hyp. Prot.

A1S_3799 632 hyp. Prot.

A1S_3805 191 hyp. Prot.

A1S_3809 176 hyp. Prot.

A1S_3835 92 hyp. Prot.

A1S_3838 92 hyp. Prot.

A1S_3851 296 hyp. Prot.

A1S_3913 347 hyp. Prot.

______________________________________________________________________
