# Supplementary material for: Molecular Mechanisms of Ethanol-Induced Pathogenesis Revealed by RNA-Sequencing
Source: PLoS Pathog. 2010 Apr 1;6(4):e1000834. doi: 10.1371/journal.ppat.1000834 (PMC2848557; doi:10.1371/journal.ppat.1000834)
Supplement: Table S3 — Oligonucleotides used in this work. (0.02 MB DOC) [file ppat.1000834.s004.doc]

**Table S3. Oligonucleotides used in this work.**

**______________________________________________________________________**

For constructs:

Name sequence Restriction site included

______________________________________________________________________

A1S_0043.1 cgcctaccagtaattgcaaaa BamHI

A1S_0043.2 tgaccaggcttaggagattctg BamHI/SalI

A1S_0043.A gtaagttgccggagcaactaac StuI

A1S_0043.B gaaatgggaattcgaccatc StuI

Kanfw1 gggaaagccacgttgtgtctc EcoRI

Kanrev1 gctgaggtctgcctcgtgaag EcoRI

Acc3 cgaattgacataagcctgttc EcoRI

Acc4 cggcgttgtgacaatttacc EcoRI

AB2448up2 gcttgcggttttagcgatta EcoRI

AB2448dw5 gctgctttgcttgagctaga PstI

______________________________________________________________________

For RT-PCR:

Name sequence gene product

______________________________________________________________________

A1S_2098RT1 cccaattactgatgcgtgtg Ethanol dehydrogenase

A1S_2098RT2 ttcacaaacatggggtagca

A1S_1266A tgccatggataaaagcatga Putative transporter protein

A1S_1266B caactgtaccgccaacaatg

A1S_2664RT1 cagcgcacaaaatcactgtt GroEL

A1S_2664RT2 tcgtctacgcggtctttctt

A1S_0294RT3 gcatgaaagaaggccaaaaa HSP90

A1S_0294RT4 aatggcgtaccgtcaaactc

A1S_0043RT1 ttgccttagcgaatgctttt phospholipase C

A1S_0043RT2 gttgatggtccaatcccatc

A1S_2846F1 gcaggtgtagttgcaggtga sulfite reductase protein

A1S_2846R2 atctcgcctgcttcattacg

A1S_0880F1 ttcccaaacccgtgataaag MinC

A1S_0880R2 gatgcggtcagagtcacgta

A1S_2578F1 gccatatgggcaaaatatcg non-ribosomal peptide synthetase

A1S_2578R2 tttccagttgttgacgatgc

A1S_2566F1 gtgaaaaattccgtgccagt siderophore receptor protein

A1S_2566R2 cattcaggcgcaaaatcata

A1S_2381F1 tggattgaaacaccagcaga acinetobactin biosynthesis protein

A1S_2381R2 gctggcagagcacacaataa

A1S_1601A ttcatggaccgtactggtga malate synthase

A1S_1601B agcatttggatgagcagctt

A1S_2710A gctcaccctatggcaatcat citrate synthase

A1S_2710B gtctgcaggagttgcaaaca

A1S_2148A caccgaattacgactgctca acetyl-CoA synthetase

A1S_2148B ctggctcacattgctcagaa

A1S_0482A tggtagctcgacttgtgcag acetate kinase

A1S_0482B tcactgtacgcatatcgttag

______________________________________________________________________
